# Nutritional Ecology, Nutritional Geometry, and Aging Research

**DOI:** 10.1093/geroni/igaa057.3102

**Published:** 2020-12-16

**Authors:** David Raubenheimer, Stephen Simpson, David Le Couteur

**Affiliations:** 1 University of Sydney, Sydney, New South Wales, Australia; 2 University of Sydney, Camperdown, New South Wales, Australia

## Abstract

Substantial advances have been made in understanding both evolutionary and mechanistic aspects of biological ageing, but the two areas remain poorly integrated. I suggest that a greater emphasis on ecology can help to integrate evolutionary and mechanistic research on ageing, by providing insight into the interface between biological mechanisms and the environments in which they evolved. Among the most salient aspects of the environment relevant to ageing is nutrition. And yet in the bulk of ageing research nutrition is coarsely represented as dietary restriction or caloric restriction, without consideration for which components of the diet or which energetic substrates are driving the observed effects. I show how a method developed in nutritional ecology, called the nutritional geometry framework, can help to understand the nutritional interactions of animals with their environments, by explicitly distinguishing the roles of calories, individual nutrients and nutrient balance. Part of a symposium sponsored by the Nutrition Interest Group.

